# Refining the criteria for immediate total-body CT after severe trauma

**DOI:** 10.1007/s00330-019-06503-2

**Published:** 2020-01-23

**Authors:** Kaij Treskes, Teun P. Saltzherr, Michael J. R. Edwards, Benn J. A. Beuker, Esther M. M. Van Lieshout, Joachim Hohmann, Jan S. K. Luitse, Ludo F. M. Beenen, Markus W. Hollmann, Marcel G. W. Dijkgraaf, J. Carel Goslings, on behalf of the REACT-2 study group, J. C. Sierink, S. van Dieren, V. M. de Jong, D. Den Hartog, T. Hagenaars, G. S. R. Muradin, R. Bingisser, C. Zähringer, N. Bless, R. van Vugt, T. N. Tromp, M. Brink, K. ten Duis, J. S. Harbers, K. W. Wendt

**Affiliations:** 1 Trauma Unit, Department of Surgery, Amsterdam University Medical Centers, location AMC, Meibergdreef 9, 1105 AZ Amsterdam, the Netherlands; 2 Department of Surgery, Haaglanden Medical Center, Lijnbaan 32, 2512 VA Den Haag, the Netherlands; 3 Trauma Unit, Department of Surgery, Radboud University Medical Center, Geert Grooteplein-Zuid 10, 6525 GA Nijmegen, the Netherlands; 4 Trauma Unit, Department of Surgery, University Medical Center Groningen, Hanzeplein 1, 9700 RB Groningen, the Netherlands; 5 Trauma Research Unit, Department of Surgery, Erasmus MC, University Medical Center Rotterdam, ’s-Gravendijkwal 230, 3015 CE Rotterdam, the Netherlands; 6 Department of Radiology and Nuclear Medicine, University of Basel Hospital, Petersgraben, 4031 Basel, Switzerland; 7 Department of Radiology, Amsterdam University Medical Centers, location AMC, Meibergdreef 9, 1105 AZ Amsterdam, the Netherlands; 8 Department of Anaesthesiology, Amsterdam University Medical Centers, location AMC, Meibergdreef 9, 1105 AZ Amsterdam, the Netherlands; 9 Clinical Research Unit/Department of Clinical Epidemiology, Biostatistics and Bioinformatics, Amsterdam University Medical Centers, location AMC, University of Amsterdam, Meibergdreef 9, 1105 AZ Amsterdam, the Netherlands; 10 Department of Surgery, Onze Lieve Vrouwe Gasthuis, Jan Tooropstraat 164, 1061 AE Amsterdam, the Netherlands

**Keywords:** Multiple trauma, Wounds and injuries, Diagnostic imaging, Multidetector computed tomography, Whole-body imaging

## Abstract

**Objectives:**

Initial trauma care could potentially be improved when conventional imaging and selective CT scanning is omitted and replaced by immediate total-body CT (iTBCT) scanning. Because of the potentially increased radiation exposure by this diagnostic approach, proper selection of the severely injured patients is mandatory.

**Methods:**

In the REACT-2 trial, severe trauma patients were randomized to iTBCT or conventional imaging and selective CT based on predefined criteria regarding compromised vital parameters, clinical suspicion of severe injuries, or high-risk trauma mechanisms in five trauma centers. By logistic regression analysis with backward selection on the 15 study inclusion criteria, a revised set of criteria was derived and subsequently tested for prediction of severe injury and shifts in radiation exposure.

**Results:**

In total, 1083 patients were enrolled with median ISS of 20 (IQR 9–29) and median GCS of 13 (IQR 3–15). Backward logistic regression resulted in a revised set consisting of nine original and one adjusted criteria. Positive predictive value improved from 76% (95% CI 74–79%) to 82% (95% CI 80–85%). Sensitivity decreased by 9% (95% CI 7–11%). The area under the receiver operating characteristics curve remained equal and was 0.80 (95% CI 0.77–0.83), original set 0.80 (95% CI 0.77–0.83). The revised set retains 8.78 mSv (95% CI 6.01–11.56) for 36% of the non-severely injured patients.

**Conclusions:**

Selection criteria for iTBCT can be reduced from 15 to 10 clinically criteria. This improves the positive predictive value for severe injury and reduces radiation exposure for less severely injured patients.

**Key Points:**

*• Selection criteria for iTBCT can be reduced to 10 clinically useful criteria.*

*• This reduces radiation exposure in 36% of less severely injured patients.*

*• Overall discriminative capacity for selection of severely injured patients remained equal.*

**Electronic supplementary material:**

The online version of this article (10.1007/s00330-019-06503-2) contains supplementary material, which is available to authorized users.

## Introduction

Improvements in speed and accuracy of computed tomography (CT) made immediate total-body CT (iTBCT) feasible as a diagnostic tool in the primary care for severe trauma patients. Initial trauma care for severe trauma patients can be improved when the step-up approach of conventional imaging and selective CT is omitted and an iTBCT is performed instead. iTBCT scanning is safe, shortens the time to end of imaging, and does not increase direct medical costs [1]. However, it has not been demonstrated to improve survival [1]. Because of the potentially increased radiation exposure by this diagnostic approach, proper selection of severely injured patients is mandatory [2–4]. Criteria for total-body CT in trauma vary across trauma centers and consensus is lacking [5, 6]. Early identification of severely injured patients will reduce exposure to radiation by iTBCT in less severely injured patients.

The decision to perform an iTBCT is based on information obtained during the pre-hospital phase and during the in-hospital primary survey. Justification for performing an iTBCT is only possible in hindsight, when radiologic imaging, interventions, and the clinical course have confirmed all diagnoses. The REACT-2 was a randomized controlled trial setup to determine the effect of iTBCT on mortality compared to conventional imaging and selective CT. Inclusion criteria of this multicenter randomized trial aimed to select severely injured patients benefitting most from iTBCT before imaging [7].

The aim of the present analysis was to assess the discriminatory power of REACT-2 criteria for severely injured patients that could benefit from iTBCT during the primary assessment of trauma care. Furthermore, a revised set of criteria was derived and tested for discriminatory characteristics on detection of severe injury and shifts in radiation exposure compared to the original set of REACT-2 inclusion criteria.

## Materials and methods

### Study design and patient selection

This study is a secondary analysis of the REACT-2 trail in which non-pregnant adult severe trauma patients were included in five trauma centers in the Netherlands and Switzerland between April 2011 and January 2014. Inclusion was based on predefined compromised vital parameters, clinical suspicion of specific severe injuries, and high-risk trauma mechanisms. Patients were considered eligible when meeting one or more of the 15 inclusion criteria and none of the exclusion criteria as shown in Table 6 in the Appendix.

Patients were randomized to iTBCT or the standard workup (STWU) that consists of conventional imaging with selective CT of specific body regions (i.e., head, neck, chest and/or abdomen, and pelvis). Decision of eligibility by the trauma leader as well as documentation of the concerning criteria by a trauma team member was performed before the start of radiologic imaging. After obtaining vital parameters, a physical examination, and potentially life-saving interventions (e.g., securing airway, chest tube placement, or hemorrhage control measures), the trauma team proceeded to CT scanning in the same or an adjacent trauma resuscitation room. CT scanning could be interrupted any moment when the patient should deteriorate and could be reached within seconds by trauma team members. iTBCT was performed without preceding conventional imaging and consisted of an unenhanced CT of the head and neck with arms alongside the trunk. The second part consisted of a contrast enhanced CT of chest, abdomen, and pelvis. The preferred technique of the second part was split-bolus intravenous contrast imaging with the arms raised alongside the head [8]. Brain reconstruction was in axial planes with 5-mm head kernel and 1-mm bone kernel, cervical spine in 1-mm bone kernel in axial, sagittal, and coronal planes. Torso was reconstructed at 3-mm axial and coronal slices in soft and bone kernel. CT scanners at the participating sites were all 64-slice multidetector row CT scanners. Indication for selective CT of specific body regions was set by local protocols.

The design of the REACT-2 study has been previously described (ClinicalTrials.gov: NCT01523626) and published [7]. The REACT-2 study was approved by the medical ethics committees at all participating centers (AMC MEC 10/145).

### Outcome

iTBCT was considered justified if a patient was classified as severely injured by in-hospital findings and clinical course. Definition of severe injury in the current study was met by presence of at least one of the following conditions:

- Injury Severity Score (ISS) ≥ 16
- Requiring emergency surgery or emergency radiologic intervention
- Direct admission to the intensive care unit
- In-hospital death

### Statistical analysis

Continuous data with a normal distribution are presented as means with standard deviation and non-normally distributed data are presented as medians with interquartile ranges. Independent sample *t* tests and Mann-Whitney *U* tests were used to compare the parametric and non-parametric continuous data, respectively. The chi-square test was used to compare the categorical variables. A *p* value of less than 0.05 was considered statistically significant.

To identify criteria that could select severely injured patients, we entered all REACT-2 inclusion criteria in backward stepwise multivariate logistic regression analysis on severe injury using *p* < 0.05 as criterion. These criteria are clinically useful and available early in the primary trauma assessment. Selection by univariate logistic regression analysis on single REACT-2 inclusion criteria before the multivariate analysis was omitted since the criteria were defined in advance. Thereby, there were more events or non-events (i.e., status as severely injured patient or status as non-severely injured patient) present in the study population than 10-fold the 15 REACT-2 inclusion criteria, which allowed multivariate analysis of all criteria. When clinically appropriate, the threshold values for vital parameters and trauma mechanism characteristics of specific criteria were retrospectively adjusted and included again in the regression analysis. Threshold value for pulse was increased by steps of 10 per minute, for systolic blood pressure (SBP) lowered by steps of 10 mmHg, and for fall from height by steps of 1 m. Positive predictive value (PPV), relative sensitivity, and receiver operating characteristics (ROC) were used to compare the accuracy of the sets of criteria.

Numbers needed to iTBCT scan to perform one unnecessary iTBCT scan for a non-severely injured patient were compared between the sets of criteria, calculated by (1/(1 − PPV)). Reduction of iTBCT scans for non-severely injured patients was calculated by subtraction of false positive rates (1 − PPV). Shifts in radiation exposure were calculated by subtraction of the sum of all effective doses from all radiological examinations done in the trauma room. The radiation dose was estimated based on the dose catalog of Mettler and colleagues [9]. Differences of the mean for radiation doses were presented with 95% CI. All statistical analyses were performed with SPSS version 24 (SPSS Inc.).

## Results

In the REACT-2 trial, 1083 patients were enrolled of which 541 (50.0%) underwent iTBCT as primary diagnostic modality. Within the entire group, 785 patients (72.5%) eventually underwent TBCT during the primary assessment as they underwent an iTBCT or CT scans from the head, neck, chest, abdomen, and pelvis secondary to x-rays and ultrasound. Median age was 43 (IQR 27–59) and 76% of the patients were male. Median ISS was 20 (IQR 9–29) and median in-hospital Glasgow Coma Scale (GCS) was 13 (IQR 3–15). Baseline demographic and clinical characteristics are presented in Table 1.

**Table 1:** Baseline demographic and clinical characteristics, *n*_max_ = 1083

Characteristic	n*	
Age (years)	1083	43 (27–59)
Male sex, *n* (%)	1083	824 (76.1)
Blunt trauma, *n* (%)	1083	1064 (98.2)
Trauma mechanism blunt trauma, *n* (%)	1064	
Fall from height		348 (32.7)
MVC—patient as occupant		391 (36.7)
MVC—patient as cyclist		125 (11.7)
MVC—patient as pedestrian		74 (7.0)
Other		126 (11.8)
Pre-hospital vital parameters		
Respiratory rate (per minute)	640	16 (14–20)
Pulse (bpm)	948	89 (25)^†^
Systolic blood pressure (mmHg)	910	133 (31)^†^
GCS (points)	1061	14 (6–15)
Triage Revised Trauma Score	618	7.04 (5.03–7.84)
In-hospital vital parameters		
Respiratory rate (per minute)	669	16 (14–20)
Pulse (bpm)	1059	88 (22)^†^
Systolic blood pressure (mmHg)	1060	131 (27)^†^
Hypotensive at admission, *n* (%)	-	82 (7.7)
GCS (points)	1083	13 (3–15)
Revised Trauma Score	651	7.11 (4.09–7.84)
Total-body CT, *n* (%)	1083	785 (72.5)
Immediate total-body CT, *n* (%)	1083	553 (51.1)
Abbreviated Injury Scale ≥ 3, *n* (%)	1083	
Head		465 (42.9)
Chest		435 (40.2)
Abdomen		116 (10.7)
Extremities		304 (28.1)
Injury Severity Score (points)	1083	20 (9–29)
Multitrauma patients, *n* (%)^‡^	1083	693 (64.0)
TBI patients, *n* (%)^‡^	1083	329 (30.4)
TRISS, survival probability	618	0.94 (0.68–0.98)

Results of the population described in this table were published earlier [1]. All data are number (%) or median (interquartile range) unless otherwise specified

*This column displays the number of patients that was analyzed for each specific variable

^†^Mean (SD)

^‡^Multitrauma patients are defined as ISS ≥ 16. TBI patients are defined as GCS < 9 at presentation and AIS head ≥ 3

*MVC* motor vehicle collision, *CT* computed tomography, *TBI* traumatic brain injury, *TRISS* Trauma and Injury Severity Score

There were 827 severely injured patients as defined by the combined outcome and therefore the original set of criteria has a PPV for severe injury of 76% (95% CI 74–79%). Table 2 presents the prevalence within the enrolled population and the PPV for each separate criterion. Backward logistic regression analysis of the 15 original criteria resulted in selection of seven criteria shown in Table 3. After adjustment of threshold values for vital parameters and trauma mechanism characteristics, the backward selection resulted in nine original and one adjusted criteria. Therefore, five of the original criteria (respiratory rate ≥ 30/min or ≤ 10/min, pulse ≥ 120/min, ejection form a vehicle, death of occupant in same vehicle, and severely injured patient in same vehicle) were not of additional value and can be omitted.

**Table 2:** Predictive value of REACT-2 immediate total-body CT criteria for severe injuries, *n* = 1083

	*n*	PPV, % (95% CI)	NPV, %* (95% CI)	Sens, %* (95% CI)	Spec, %* (95% CI)
Parameters at hospital arrival					
Respiratory rate ≥ 30/min or ≤ 10/min	16	81 (62–100)	24 (21–26)	2 (1–2)	99 (98–100)
Pulse ≥ 120/min	69	80 (70–89)	24 (21–27)	7 (5–8)	95 (92–97)
Pulse ≥ 130/min^†^	49	88 (79–97)	24 (22–27)	5 (4–7)	98 (96–100)
Pulse ≥ 140/min^†^	26	88 (63–76)	24 (21–27)	3 (2–4)	99 (98–100)
Systolic blood pressure ≤ 100 mmHg	116	96 (92–99)	26 (23–29)	13 (11–16)	98 (96–100)
Systolic blood pressure < 90 mmHg^†^	82	100 (100–100)	26 (23–28)	10 (8–12)	100 (100–100)
Systolic blood pressure < 80 mmHg^†^	32	100 (100–100)	24 (22–27)	4 (3–5)	100 (100–100)
Estimated exterior blood loss ≥ 500 ml	43	91 (82–99)	24 (22–27)	5 (3–6)	98 (97–100)
GCS ≤ 13 or abnormal pupillary reaction	485	93 (91–95)	37 (33–41)	55 (51–58)	87 (83–91)
GCS ≤ 8^†^	437	99 (98–100)	39 (35–43)	52 (49–56)	98 (97–100)
GCS = 3^†^	394	99 (99–100)	37 (33–41)	47 (44–51)	99 (98–100)
Clinical suspicions					
Fractures from at least two long bones	90	89 (82–95)	25 (22–28)	10 (8–12)	96 (94–99)
Flail chest, open chest, or multiple rib fractures	114	83 (76–90)	25 (22–27)	12 (9–14)	93 (89–96)
Severe abdominal injury	65	82 (72–91)	24 (21–27)	6 (5–8)	95 (93–98)
Pelvic fracture	98	78 (69–86)	24 (21–27)	9 (7–11)	91 (88–95)
Unstable vertebral fractures/spinal cord compression	69	68 (57–79)	23 (21–26)	6 (4–7)	91 (88–95)
Injury mechanisms					
Fall from height (> 3 m/> 10 ft)	319	62 (57–67)	18 (15–20)	24 (21–27)	53 (47–59)
Fall from height (> 4 m/> 13 ft)^†^	166	70 (64–77)	23 (20–25)	14 (12–17)	81 (76–86)
Fall from height (> 5 m/> 16 ft)^†^	126	71 (64–79)	23 (20–26)	11 (9–13)	86 (82–90)
Fall from height (> 6 m/> 20 ft)^†^	82	78 (69–87)	24 (21–26)	8 (6–10)	93 (90–96)
Fall from height (> 7 m/> 23 ft)^†^	60	87 (78–95)	24 (22–27)	6 (5–8)	97 (95–99)
Fall from height (> 8 m/> 26 ft)^†^	40	88 (77–98)	24 (22–27)	4 (3–6)	98 (96–100)
Ejection from a vehicle	30	60 (42–78)	23 (21–26)	2 (1–3)	95 (93–98)
Death of occupant in same vehicle	17	65 (42–87)	24 (21–26)	1 (1–2)	98 (96–100)
Severely injured patient in same vehicle	18	78 (59–97)	24 (21–26)	2 (1–3)	98 (97–100)
Wedged or trapped chest/abdomen	60	83 (74–93)	24 (21–27)	6 (4–8)	96 (94–99)

*Within the group of patients selected by the original criteria

^†^Retrospectively adjusted criteria

*GCS* Glasgow Coma Scale, *PPV* positive predictive value, NPV negative predictive value, *Sens* sensitivity, *Spec* specificity, *CI* confidence interval

**Table 3:** Predictive value of REACT**-**2 immediate total-body CT criteria for severe injuries, *n* = 1083

				Backward selection of criteria			
		Univariate analysis		Original criteria		Adjusted criteria	
	*n*	OR (95% CI)	*p*	OR (95% CI)	*p*	OR (95% CI)	*p*
Parameters at hospital arrival							
Respiratory rate ≥ 30/min or ≤ 10/min	16	1.35 (0.38–4.76)	0.644	–	–	–	–
Pulse ≥ 120/min	69	1.23 (0.67–2.25)	0.499	–	–	–	–
Pulse ≥ 130/min*	49	2.29 (0.96–5.43)	0.061			–	–
Pulse ≥ 140/min*	26	2.41 (0.72–8.10)	0.154			–	–
Pulse (continuous)^†^		1.02 (1.01–1.02)	< 0.001			–	–
Systolic blood pressure ≤ 100 mmHg	116	7.78 (3.14–19.29)	< 0.001	5.71 (2.23–14.62)	< 0.001	5.72 (2.22–14.75)	< 0.001
Systolic blood pressure < 90 mmHg*	82	∞ (0–∞)	0.996				
Systolic blood pressure < 80 mmHg*	32	∞ (0–∞)	0.998				
Systolic blood pressure (continuous)^†^		0.99 (0.98–0.99)	< 0.001				
Estimated exterior blood loss ≥ 500 ml	43	3.12 (1.10–8.81)	0.032	3.29 (1.09–9.93)	0.035	3.70 (1.20–11.37)	0.023
GCS ≤ 13 or abnormal pupillary reaction	485	7.83 (5.32–11.52)	< 0.001	10.02 (6.69–15.00)	< 0.001	12.65 (8.23–19.45)	< 0.001
GCS ≤ 8*	437	69.24 (25.54–187.66)	< 0.001				
GCS = 3*	394	114.45 (28.28–463.28)	< 0.001				
GCS (continuous)^†^		0.69 (0.64–0.74)	< 0.001				
Clinical suspicions							
Fractures from at least two long bones	90	2.64 (1.34–5.16)	0.005	4.08 (2.02–8.25)	< 0.001	4.94 (2.41–10.15)	< 0.001
Flail chest, open chest, or multiple rib fractures	114	1.62 (0.97–2.71)	0.066	2.81 (1.62–4.86)	< 0.001	3.27 (1.85–5.76)	< 0.001
Severe abdominal injury	65	1.39 (0.73–2.65)	0.313	–	–	2.18 (1.07–4.42)	0.031
Pelvic fracture	98	1.08 (0.66–1.77)	0.771	1.76 (1.03–3.01)	0.039	1.82 (1.05–3.14)	0.033
Unstable vertebral fractures/spinal cord compression	69	0.64 (0.38–1.09)	0.098	–	–	1.87 (1.06–3.31)	0.032
Injury mechanisms							
Fall from height (> 3 m/> 10 ft)	319	0.35 (0.26–0.47)	< 0.001	–	–	–	–
Fall from height (> 4 m/> 13 ft)*	166	0.70 (0.48–1.01)	0.054			1.64 (1.07–2.52)	0.022
Fall from height (> 5 m/> 16 ft)*	126	0.75 (0.49–1.13)	0.167				
Fall from height (> 6 m/> 20 ft)*	82	1.11 (0.65–1.91)	0.709				
Fall from height (> 7 m/> 23 ft) *	60	2.08 (0.98–4.44)	0.058				
Fall from height (> 8 m/> 26 ft)*	40	2.22 (0.86–5.72)	0.099				
Fall from height (continuous)^†^		1.17 (1.06–1.29)	0.002				
Ejection from a vehicle	30	0.45 (0.22–0.95)	0.037	–	–	–	–
Death of occupant in same vehicle	17	0.56 (0.21–1.53)	0.261	–	–	–	–
Severely injured patient in same vehicle	18	1.09 (0.35–3.33)	0.887	–	–	–	–
Wedged or trapped chest/abdomen	60	2.71 (1.04–4.54)	0.038	2.11 (1.00–4.42)	0.049	2.57 (1.20–5.51)	0.015

*Retrospectively adjusted criteria

^†^Continuous data of regarding criterion used were possible

*GCS* Glasgow Coma Scale, *OR* odds ratio, *CI* confidence interval

Table 4 shows that PPV of the newly formed set of criteria statistically significantly increased to 82% (95% CI 80–85%) compared to 76% (95% CI 74–79%) of the original set. Sensitivity of the revised set within the originally formed population was statistically significantly reduced by 9% (95% CI 7–11%). Clinical characteristics including trauma scores comparing severely injured patients not selected by the revised set of criteria to selected severely injured patients are displayed in Table 8 in the Appendix. The area under the ROC curve remained equal and was 0.80 (95% CI 0.77–0.83) in the revised set compared to 0.80 (95% CI 0.77–0.83) for the original set as shown in Figure 1 in the Appendix. Numbers of iTBCT scans needed to perform one unnecessary scan for a non-severely injured patient was statistically significantly improved from 1 in 4.2 (95% CI 3.8–4.7) to 1 in 5.6 (95% CI 4.9–6.5). The number of unnecessary iTBCT scans was statistically significantly decreased with 6% (95% CI 2–10%).

**Table 4:** Characteristics of different sets of criteria for immediate total-body CT

	PPV (95% CI)	Relative sensitivity* (95% CI)	ROC AUC (95% CI)	Numbers needed to overscan^†^ (95% CI)	Decrease of unnecessary iTBCT scans^‡^ (95% CI)
Original criteria (*n* = 15)	76% (74–79)	Reference	0.80 (0.77–0.83)	4.2 (3.8–4.7)	Reference
Selected original criteria (*n* = 7)	87% (85–90)	80% (77–83)	0.78 (0.75–0.81)	7.9 (6.7–9.8)	11% (8–14)
Selected adjusted criteria (*n* = 10)	82% (80–85)	91% (89–93)	0.80 (0.77–0.83)	5.6 (4.9–6.5)	6% (2–10)

*Relative sensitivity within the population preselected by the original criteria

^†^Number of iTBCT scans to perform one unnecessary iTBCT for a non-severely injured patient

^‡^Percentage decrease of iTBCT scans for non-severely injured patients

*ROC* receiver operating characteristic, *AUC* area under the curve, *PPV* positive predictive value, *CI* confidence interval

Shifts in radiation exposure for the different sets of criteria are displayed separately for severely injured and non-severely injured patients in Table 5. With the use of the original criteria, iTBCT adds 1.19 mSv (95% CI − 0.13–2.51) for severely injured patients and 8.15 mSv (95% CI 5.91–10.39) for non-severely injured patients compared to the STWU. Within patients not selected for iTBCT by the revised criteria, the STWU retains 1.32 mSv (95% CI − 2.71–5.35) for 9% of the severely injured patients and retains 8.78 mSv (95% CI 6.01–11.56) for 36% of the non-severely injured patients compared to iTBCT. Shifts in radiation exposure are displayed separately for age groups < 45 years and > 45 years in Tables 8 and 9 in the Appendix.

**Table 5:** Shifts in radiation exposure in different sets of criteria for immediate total-body CT and standard workup with selective CT, *n* = 1083

	Original criteria (15)		Selected criteria (7)		Selected and adjusted criteria (10)	
	Additional radiation exposure compared to STWU, mSv (95% CI)	% of population	Additional radiation exposure compared to STWU, mSv (95% CI)	% of population	Additional radiation exposure compared to STWU, mSv (95% CI)	% of population
Selected for iTBCT						
Severely injured	1.19 (− 0.13 to 2.51)	76.4	2.05 (0.56 to 3.53)	60.9	1.17 (− 0.23 to 2.57)	69.7
Non-severely injured	8.15 (5.91 to 10.39)	23.6	7.24 (3.07 to 11.41)	8.8	7.91 (4.77 to 11.05)	15.1
	Additional radiation exposure compared to iTBCT, mSv (95%CI)	% of population	Additional radiation exposure compared to iTBCT, mSv (95% CI)	% of population	Additional radiation exposure compared to iTBCT, mSv (95% CI)	% of population
Selected for STWU						
Severely injured	–	–	2.11 (− 0.74 to 4.95)	15.4	− 1.32 (− 5.35 to 2.71)	6.7
Non-severely injured	–	–	− 8.78 (− 11.44 to − 6.13)	14.9	− 8.78 (− 11.56 to − 6.01)	8.5

*iTBCT* immediate total-body CT, *STWU* standard workup with selective CT

## Discussion

By retrospective analysis of a prospectively formed cohort of severe trauma patients, we derived a revised set of 10 criteria for iTBCT, shown in Table 6. The new set of criteria has an increased PPV for detecting severe injury. Hence, these criteria could reduce the number of patients screened by iTBCT who are less severely injured and who will not have an advantage of all their body regions scanned. The relative reduction of sensitivity compared to the original set could be restrained to 9%. This reduction of sensitivity leads to a relative increase of severely injured patients for whom screening by iTBCT will be retained and will have conventional imaging and selective CT scanning. Since there is no reduction of mortality after iTBCT for the trial population selected by the original criteria, the aim for a revised set of iTBCT criteria with higher PPV and lower sensitivity can be justified. Without loss of overall discriminative capacity for severe injuries, we changed the set of criteria for iTBCT with emphasis on the reduction of radiation exposure for the less severely injured patient.

**Table 6:** Revised criteria for immediate total-body CT in trauma patients

Trauma patients with one of the following parameters at hospital arrival:	
• Systolic blood pressure < 100 mmHg	
• Estimated exterior blood loss ≥ 500 ml	
• Glasgow Coma Score ≤ 13 or abnormal pupillary reaction	
AND/OR	
Patients with a clinical suspicion of one of the following diagnoses:	
• Fractures from at least two long bones	
• Flail chest, open chest, or multiple rib fractures	
• Severe abdominal injury	
• Pelvic fracture	
• Unstable vertebral fractures/spinal cord compression	
AND/OR	
Patients with one of the following injury mechanisms:	
• Fall from a height (> 4 m/> 13 ft)	
• Wedged or trapped chest/abdomen	
Contra indications*	
Trauma patients with one of the following characteristics:	
• Known age < 18 years	
• Known pregnancy	
• Referred from another hospital	
• Clearly low-energy trauma with blunt injury mechanism	
• Any patient with a stab wound in one body region	
• Any patient who is judged to be too unstable to undergo a CT scan and requires (cardiopulmonary) resuscitation or immediate operation because death is imminent	

*Contra indications for immediate total-body CT were not revised. These criteria are mentioned in this table to give a complete overview

Quantification of the shifts in radiation exposure was performed separately for the less severely injured patients. For 36% of the less severely injured patient, a significant reduction in radiation exposure could be demonstrated by use of the revised set of criteria. This effect was also present for patients of age < 45 years. The precise amounts of reduction in radiation exposure have to be interpreted in perspective of ongoing developments of low-dose CT scanning.

Compromised vital parameters, clinical suspicion of severe injuries, and high-risk mechanisms are widely used as criteria for TBCT in severe trauma [5, 6]. The first report by Wurmb et al on such a set of criteria for iTBCT described a PPV of 69% and sensitivity of 97% for ISS ≥ 16 in sedated and ventilated severe trauma patients. The difference in outcome measure and the selection of sedated and ventilated patients makes the results difficult to compare to our study [10]. Hsiao et al reported 32% PPV and 50% sensitivity of criteria for TBCT by clinical judgment for the presence of multi-region injury defined by an Abbreviated Injury Score (AIS) of ≥ 2 in two or more body regions. After retrospective identification of predictors for multi-region injury, a prediction model was made that did not show improvement for the area under the ROC curve compared to indication by clinical judgment [11].

Hemodynamically compromised patients could benefit from trauma screening by iTBCT. Wada et al [12] reported reduced mortality for patients receiving TBCT before emergency bleeding control measurements in a retrospective study in two trauma centers. Reduction in mortality in trauma patients requiring emergency bleeding control interventions by iTBCT could not be confirmed in the REACT-2 population. However, a potentially clinically relevant absolute risk reduction of 11.2% (95% CI − 0.3 to 22.7%) in comparison with the STWU was observed [13]. Huber-Wagner et al [14] reported reduced mortality in severe trauma patients in moderate (SBP 90–110 mmHg) or severe (SBP < 90 mmHg) shock when receiving TBCT during the resuscitation in a retrospective multicenter study. In the present study, compromised blood pressure (SBP < 100 mmHg) is an independent predictor for severe injury and is therefore a valid indication for iTBCT. It is recommendable to only perform CT scanning on hemodynamically compromised patients in the trauma resuscitation room or the adjacent room and the trauma team has direct access to the patient and has options for potential life-saving interventions any moment.

Patients with a compromised GCS could benefit from trauma screening by TBCT. Kimura et al [15] reported reduced mortality in patients with moderate to severe consciousness disturbance (GCS 3–12) in a retrospective multicenter study. Furthermore, decreased levels of consciousness could be considered an indication on itself since several clinical indicators for imaging are unreliable owing to the lack of subjective input from the patient when screening for injuries. Routine CT imaging for patients with unreliable physical examination is reported to reveal unsuspected findings in up to 38%, leading to treatment changes in 19–26% [16, 17]. Our study found GCS ≤ 13 or abnormal pupillary reaction an independent predictor for severe injury and further supports a compromised GCS to be a valid indication for iTBCT after severe trauma.

Besides vital parameters that indicate a hemodynamically or neurologically compromised status, also clinical suspicions of specific injuries and high-risk trauma mechanisms independently predict patients to be severely injured in our study. Although these criteria are prone to interpretation differences, we would recommend adopting these criteria in iTBCT indication schemes. During mass casualty accidents, overruling the iTBCT indication scheme has to be considered [18, 19]. Furthermore, there should be awareness for the increase of incidental findings by TBCT compared to the STWU during implementation or refining of iTBCT indication schemes [20, 21].

### Limitations and strengths

The main limitation of this study is the lack of information of patients who were not selected by the original REACT-2 criteria for eligibility of screening by iTBCT. This study could therefore only report the relative reduction of the sensitivity by the revised set compared to the original set of criteria. If proportions of severely injured patients in the group not selected by the original criteria were available, the absolute sensitivity, specificity, and negative predictive value could have been calculated. The proposed revised set of iTBCT criteria should be prospectively validated in another cohort of patients.

The definition of multitrauma and multi-region injured patients is subject of debate. Several cut-off values for ISS or AIS are used with eventual involvement of vital parameters proposed [22]. As a part of the combined outcome measure of this study, we chose ISS ≥ 16 to justify iTBCT in hindsight for patients with multiple relevant injuries (AIS ≥ 3 in two or more body regions or AIS ≥ 3 in one body region and AIS ≥ 2 in two or more body regions) and patients with a severe injury of at least one body region (AIS ≥ 4). Hsiao et al [11] chose AIS ≥ 2 in two regions as the anatomical outcome measure to justify TBCT. In our opinion, TBCT for patients with eventually AIS of 2 in two body regions is not justified. On the contrary, the screening of a patient with a severe injury in only one body region could be justified since there is a higher probability of concomitant injury, which should be quickly excluded with high accuracy.

An alternative approach for refining the criteria for iTBCT criteria is to determine its discriminative power for selection of patients who would otherwise receive equal or even higher radiation exposure by selective CT scanning compared to the radiation exposure of iTBCT. This particularly reflects the judgment of the trauma team leader for the necessity of CT scans of specific body regions which does not necessarily correlates with selection of severely injured patients [23]. Therefore, the radiation exposure by the diagnostic approach with selective CT scans was not eligible as outcome measure for revision of the iTBCT criteria.

Strength of this multicenter study is the assessment of prospectively observed criteria for iTBCT in a large trial population. Previous studies assessed retrospectively observed TBCT criteria or were performed in a single-center setting. The combined clinical outcome parameter is suitable to define severely injured patients and patients that need fast and detailed diagnostics when an immediate intervention or ICU treatment is indicated. The addition of immediate surgery to the combined outcome measure is supported by reports of potential time and survival benefit for patients receiving emergency surgery [12, 24]. The revised set of criteria will reduce the exposure to radiation for less severely injured patients without loss of discriminative capacity for severe injury. Thereby, the revision led to a simplification, which implies easier application during primary trauma care.

## Conclusion

This study presents a revised set of 10 clinically criteria for iTBCT with a high predictive value for severe injury and therefore reduces radiation for the less severely injured patients for iTBCT. The criteria selected as predictors in this study should be prospectively validated in another cohort of patients for whom screening by iTBCT is considered after severe trauma.

## Electronic supplementary material

ESM 1 (DOCX 161 kb)

## Abbreviations

AIS: Abbreviated injury score
CI: Confidence interval
CT: Computed tomography
GCS: Glasgow coma scale
IQR: Interquartile range
ISS: Injury severity score
iTBCT: Immediate total-body CT
PPV: Positive predictive value
ROC: Receiver operating characteristics
SBP: Systolic blood pressure
STWU: Standard workup
