# Supplementary material for: Refining the criteria for immediate total-body CT after severe trauma
Source: Eur Radiol. 2020 Jan 23;30(5):2955–63. doi: 10.1007/s00330-019-06503-2 (PMC7160085; doi:10.1007/s00330-019-06503-2)
Supplement: Supplementary file 1 — (DOCX 161 kb) [file 330_2019_6503_MOESM1_ESM.docx]

**Supplementary Materials**

**Figure 1.** Receiver operating characteristic (ROC) curves for severe injury of the original criteria, selected criteria and the selected adjusted criteria

**Appendix table 7.** Original indications for immediate total-body CT in trauma patients used in REACT-2 trial

Trauma patients with one of the following parameters at hospital arrival:

- respiratory rate ≥30/min or ≤10/min
- pulse ≥120/min
- systolic blood pressure ≤100 mmHg
- estimated exterior blood loss ≥500 ml
- Glasgow Coma Score ≤13 or abnormal pupillary reaction

AND / OR

Patients with a clinical suspicion of one of the following diagnoses:

- fractures from at least two long bones
- flail chest, open chest or multiple rib fractures
- severe abdominal injury
- pelvic fracture
- unstable vertebral fractures / spinal cord compression

AND / OR

Patients with one of the following injury mechanisms:

- fall from a height (>3 meters / >10 feet)
- ejection from a vehicle
- death of occupant in same vehicle
- severely injured patient in same vehicle
- wedged or trapped chest / abdomen

**Contra indications**

Trauma patients with one of the following characteristics were excluded:

- known age <18 years
- known pregnancy
- referred from another hospital
- clearly low-energy trauma with blunt injury mechanism
- any patient with a stab wound in one body region
- any patient who is judged to be too unstable to undergo a CT scan and requires (cardiopulmonary) resuscitation or immediate operation because death is imminent

| **Table 8.** Clinical characteristics for severely injured patients not selected by the revised set of criteria (n=75) in comparison to selected severely injured patients (n=752). | | | | |
| --- | --- | --- | --- | --- |
| Characteristic | Not selected (n)  vs.  selected (n)* | Not selected  (n=75) | Selected  (n=752) | P-value |
| Age (years) | 75 vs. 752 | 47 (30-59) | 43 (26-60) | 0.279^†^ |
| Male sex, n (%) | 75 vs. 752 | 60 (80.0) | 571 (75.9) | 0.429^‡^ |
| Blunt trauma, n (%) | 75 vs. 752 | 71 (94.7) | 741 (98.5) | 0.039^‡^ |
| Abbreviated Injury Scale ≥3, n (%)  Head  Chest  Abdomen  Extremities | 75 vs. 752 | 37 (49.3)  33 (44.0)  9 (12.0)  19 (25.3) | 415 (55.2)  388 (51.6)  107 (14.2)  253 (33.6) | 0.332^‡^  0.210^‡^  0.596^§^  0.144^‡^ |
| Injury Severity Score (points)  Polytrauma patients, n (%)\|\| | 75 vs. 752  75 vs. 752 | 22 (17-29)  60 (80.0) | 25 (17-34)  633 (84.2) | 0.009^†^  0.349^‡^ |
| TRISS, survival probability | 31 vs. 459 | 0.94 (0.85-0.98) | 0.88 (0.51-0.97) | 0.031^†^ |
| In-hospital mortality, n (%) | 75 vs. 752 | 10 (13.3) | 161 (21.4) | 0.133^§^ |
| Length of stay (days)  Total hospital stay  ICU stay  Ventilation days | 75 vs. 752  75 vs. 752  75 vs. 752 | 11 (4-19)  1 (0-2)  0 (0-1) | 11 (4-22)  1 (0-5)  1 (0-3) | 0.124^†^  0.249^†^  0.059^†^ |

All data are number (%) or median (interquartile range) unless otherwise specified.

* This column displays the number of patients that was analysed for each specific variable.

† Mann-Whitney U test; ‡Chi^2^ test; §Fisher’s Exact Test.

||Polytrauma patients are defined as ISS ≥16. Traumatic Brain Injury (TBI) patients are defined as GCS <9 at presentation and AIS Head ≥3.

TRISS denotes Trauma and Injury Severity Score.

| **Table 9.** Shifts in radiation exposure in different sets of criteria for immediate total body CT and standard work-up with selective CT for patients of age < 45 years, n= 562. | | | | | | | | | |
| --- | --- | --- | --- | --- | --- | --- | --- | --- | --- |
|  |  | Original criteria (15) | |  | Selected criteria (7) | |  | Selected and adjusted criteria (10) | |
|  |  | *additional radiation exposure compared to STWU, mSv (95%CI)* | *% of population* |  | *additional radiation exposure compared to STWU, mSv (95%CI)* | *% of population* |  | *additional radiation exposure compared to STWU, mSv (95%CI)* | *% of population* |
| **Selected for iTBCT** | |  |  |  |  |  |  |  |  |
| Severely injured | | 0.66 (-0.35 to 2.66) | 75.6 |  | 2.29 (0.11 to 4.47) | 61.2 |  | 1.00 (-1.09 to 3.09) | 70.3 |
| Non-severely injured | | 9.87 (6.92 to 12.82) | 24.4 |  | 9.31 (3.21 to 15.41) | 8.9 |  | 9.52 (5.30 to 13.74) | 15.7 |
|  | |  |  |  |  |  |  |  |  |
|  | | *additional radiation exposure compared to iTBCT, mSv (95%CI)* |  |  | *additional radiation exposure compared to iTBCT, mSv (95%CI)* |  |  | *additional radiation exposure compared to iTBCT, mSv (95%CI)* |  |
| **Selected for STWU** | |  |  |  |  |  |  |  |  |
| Severely injured | | - | - |  | 6.18 (1.38 to 10.98) | 14.4 |  | 3.82 (-3.69 to 11.32) | 5.3 |
| Non-severely injured | | - | - |  | -10.35 (-13.50 to -7.20) | 15.5 |  | -10.21 (-13.40 to -7.03) | 8.7 |

iTBCT denotes immediate total body CT; STWU denotes standard work-up with selective CT

| **Table 10.** Shifts in radiation exposure in different sets of criteria for immediate total body CT and standard work-up with selective CT for patients of age > 45 years, n= 521. | | | | | | | | | |
| --- | --- | --- | --- | --- | --- | --- | --- | --- | --- |
|  |  | Original criteria (15) | |  | Selected criteria (7) | |  | Selected and adjusted criteria (10) | |
|  |  | *additional radiation exposure compared to STWU, mSv (95%CI)* | *% of population* |  | *additional radiation exposure compared to STWU, mSv (95%CI)* | *% of population* |  | *additional radiation exposure compared to STWU, mSv (95%CI)* | *% of population* |
| **Selected for iTBCT** | |  |  |  |  |  |  |  |  |
| Severely injured | | 1.57 (-0.11 to 3.26) | 77.2 |  | 1.65 (-0.36 to 3.66) | 60.7 |  | 1.20 (-0.62 to 3.03) | 69.1 |
| Non-severely injured | | 6.25 (2.80 to 9.71) | 22.8 |  | 4.66 (0.27 to 9.05) | 8.6 |  | 6.12 (1.62 to 10.61) | 14.6 |
|  | |  |  |  |  |  |  |  |  |
|  | | *additional radiation exposure compared to iTBCT, mSv (95%CI)* |  |  | *additional radiation exposure compared to iTBCT, mSv (95%CI)* |  |  | *additional radiation exposure compared to iTBCT, mSv (95%CI)* |  |
| **Selected for STWU** | |  |  |  |  |  |  |  |  |
| Severely injured | | - | - |  | -1.34 (-4.20 to 1.52) | 16.5 |  | -4.66 (-8.81 to 0.51) | 8.1 |
| Non-severely injured | | - | - |  | -7.00 (-11.43 to 2.57) | 14.2 |  | -6.92 (-11.69 to 2.14) | 8.3 |

iTBCT denotes immediate total body CT; STWU denotes standard work-up with selective CT
